# Engineered microbes report levels of freshwater contamination in real time

**DOI:** 10.1093/synbio/ysad002

**Published:** 2023-02-16

**Authors:** Tea Crnković

**Affiliations:** Department of Biological Engineering, Massachusetts Institute of Technology, Cambridge, MA, USA

Human activity negatively affects freshwater ecosystems through contaminants such as pharmaceuticals, pesticides, or heavy metals, which can leach into natural water sources. Those contaminations do affect not only whatever lives in the water—animals, fish, and microbes—but also individuals who drink the water, posing a threat to millions of people who rely on limited water sources (1). As the levels of contaminant concentrations can change over time, frequent and continuous on-site monitoring would be ideal in order to be able to take appropriate public health actions.

Synthetic biologists have extensively studied biosensors for contaminant monitoring (2), especially biosensors based on living cells (i.e. bacteria), since they can be grown in simple and cheap culture media, do not require extensive sample preparation and can be field-deployed by untrained users. But two challenges remained for making these biosensors field-ready: first, existing biosensors are typically transcription-activated, limiting their response time to up to half an hour. Second, materials commonly used to encapsulate biosensing microbes, like hydrogels, further delay and attenuate responses, limiting their practical applications. These issues were recently addressed by Prof. Caroline Ajo-Franklin and her team from Rice University in Houston, Texas, by programming microbes to utilize transcription-independent electron transfer (ET) from contaminants to an electrode and by employing electroconductive nanoparticles within encapsulation material (3). These advances could launch microbial biosensors as sensitive and accurate freshwater surveillance tools, especially in contamination-prone resource-limited areas where they are most needed to provide safe drinking water.

The authors first engineered a synthetic ET pathway in *Escherichia coli* to detect thiosulfate, a dechlorination substance that in high quantities leads to excessive microbial growth in freshwater systems. In their system, thiosulfate is initially oxidized to sulfite by endogenous proteins. The sulfite then serves as an electron donor in the downstream three-module ET pathway. First, the input module reduces sulfite to sulfide and oxidizes NADPH to NADP^+^ via three cytosolic proteins: ferredoxin-NADP^+^ reductase, ferredoxin, and sulfite reductase. Next, in the coupling module, sulfide-quinone reductase oxidizes the sulfide from the input module and reduces quinones within the inner membrane into quinols. The last output module oxidizes quinols in the periplasm by quinol dehydrogenase and transfers electrons from cytochrome c–porin complex (MtrCAB) across the outer membrane to the electrode. The authors were able to detect thiosulfate at high micromolar concentrations within minutes after further optimization of the ET pathway.

Thiosulfate is just one of many potential contaminants that occur in the environment. For that reason, the authors decided to repurpose their synthetic ET pathway to detect 4-hydroxytamoxifen (4-HT), an estrogen antagonist causing reproductive dysfunction. 4-HT biosensing was achieved by replacing the native ferredoxin from the input module with a split ferredoxin bound to an estrogen receptor ligand–binding domain. By binding 4-HT to the fusion protein, a functional ferredoxin is formed, enabling complete ET from NADPH to the electrode, allowing the detection of 4-HT within minutes at low micromolar concentrations.

Next, the authors tested the robustness of their bioelectronic sensors: freshwater from different regions differs in its physical and chemical compositions—due to this, we can taste a difference in tap water from our hometown or from abroad—thus causing potential interference in electronic biosensing. Despite this, the authors detected low millimolar thiosulfate concentration in all water samples within 7 min, demonstrating the biosensor’s sustaining performance in complex samples. Still, the authors further incorporated TiO_2_@TiN nanoparticles in their microbial encapsulation material in order to increase the contact surface and facilitate ET between engineered bacteria and the electrode. Indeed, such hybrid nanoparticle–bioelectronic sensors demonstrated an improved signal-to-noise ratio at lower thiosulfate and 4-HT concentrations, as well as faster response time.

This living bioelectronic sensor coupled with conductive nanomaterials represents a significant advancement in the field of synthetic biology and contaminant detection: it can detect analytes in complex water samples with a response time limited only by the diffusion rate of the analytes through the encapsulation material. The biosensor design is a modular that can also be used to detect other molecules of interest, provided that those molecules can serve as ligands of a split ligand–binding domain.

In order to further refine the biosensor for real-world applications, the natural concentrations of contaminants have to be considered, since some can cause toxic effects at very low (sub)nanomolar concentrations (4, 5). The current detection limit of the biosensor could be improved by engineering extracellular ET proteins to function as protein switches, such that analytes would not need to enter the cell, or via the incorporation of signal amplification cascades. Ultimately, for real-world implementation of living engineered microbes for water quality surveillance, the use of well-studied robust biocontainment strategies is essential in order to prevent the release of genetically modified organisms into the environment (6).
